# Ionic scattering factors of atoms that compose biological molecules

**DOI:** 10.1107/S2052252518005237

**Published:** 2018-04-27

**Authors:** Koji Yonekura, Rei Matsuoka, Yoshiki Yamashita, Tsutomu Yamane, Mitsunori Ikeguchi, Akinori Kidera, Saori Maki-Yonekura

**Affiliations:** a Biostructural Mechanism Laboratory, RIKEN SPring-8 Center, 1-1-1 Kouto, Sayo, Hyogo 679-5148, Japan; b Computational Life Science Laboratory, Graduate School of Medical Life Science, Yokohama City University, 1-7-29, Suehiro-cho, Tsurumi-ku, Yokohama, Kanagawa 230-0045, Japan

**Keywords:** form factors, electron crystallography, single-particle cryo-EM, structure refinement, X-ray crystallography, imaging, structure determination

## Abstract

Ionic scattering factors of atoms that compose biological molecules have been computed by the multi-configuration Dirac–Fock method. These ions are chemically unstable and their scattering factors had not been reported except for O^−^. Yet these factors are required for the estimation of partial charges in protein molecules and nucleic acids. The electron scattering factors of these ions are particularly important as the electron scattering curves vary considerably between neutral and charged atoms in the spatial-resolution range explored in structural biology. The calculated X-ray and electron scattering factors have then been parameterized for the major scattering curve models used in X-ray and electron protein crystallography and single-particle cryo-EM. The X-ray and electron scattering factors and the fitting parameters are presented for future reference.

## Abstract 2

Ionic scattering factors of atoms that compose biological molecules have been computed by the multi-configuration Dirac–Fock method and parameterized for major curve models in X-ray and electron protein crystallography and single-particle cryo-EM.

## 1. Introduction   

**Figure 1:**
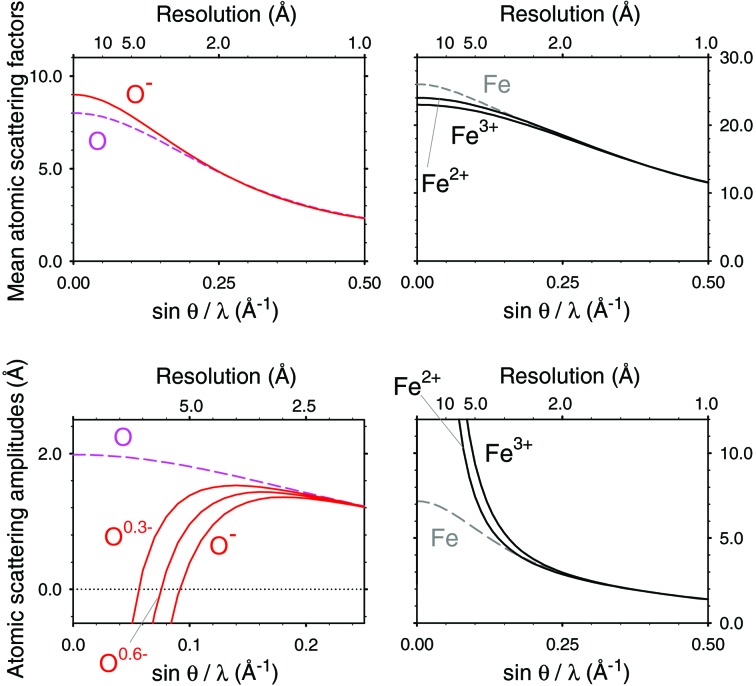
Plots of typical X-ray (upper row) and electron (lower row) scattering factors from the literature [*e.g. International Tables for Crystallography* (Colliex *et al.*, 2006 ▸)]. The electron scattering factors of O and O^−^ are displayed up to sin*θ*/*λ* = 0.25 Å^−1^ and the others are up to 0.5 Å^−1^. Electron scattering factors of partially charged O^0.3−^ and O^0.6−^ were calculated from a linear combination of those of the neutral and fully ionized oxygen atoms (Yonekura & Maki-Yonekura, 2016 ▸) and are also overlaid in the lower left graph.

Scattering factors for X-rays and electrons are essential for the analysis of experimental data probed with an X-ray and an electron beam. X-rays are scattered by electrons around the atom, yielding an electron-density map, whereas electrons are scattered by Coulomb potential, yielding a Coulomb-potential map. Hence the scattering factors are directly related to the charged state, particularly the electron scattering factors, which vary considerably between the neutral and charged atoms in the regime when sin*θ*/*λ* is smaller than 0.1 Å^−1^ and do not match until sin*θ*/*λ* reaches 0.2–0.3 Å^−1^ (Fig. 1 ▸). Here *θ* represents half the scattering angle, *λ* is the wavelength of the incident X-ray or electrons and the latter values of sin*θ*/*λ* correspond to spatial resolutions of 1/(0.2 × 2) – 1/(0.3 × 2) = 2.5 – 1.67 Å. Thus, the charge state essentially affects the structure analysis of proteins and protein complexes including nucleic acids by electron crystallography and single-particle cryo-EM.

**Figure 2:**
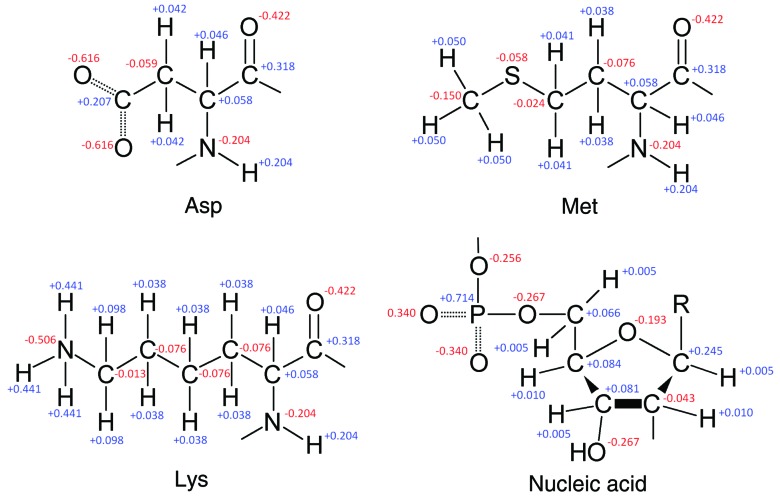
Theoretical charge distributions over atoms in typical amino acids and the template of a nucleic acid. Taken from the crystallographic information files (Brown & McMahon, 2002 ▸) in the *PHENIX* library (Adams *et al.*, 2010 ▸).

The charge is actually delocalized over several atoms in the protein molecules and nucleic acids, yielding partial charges which can be regarded as effective scattering factors. Partial charges should be assigned to all of the atoms in proteins and protein complexes (see Fig. 2 ▸ for theoretical partial charge distributions in amino acids and a nucleic acid). Although this approach is more qualitative than using form factors representing chemical bonding calculated for typical small molecules (Chang *et al.*, 1999 ▸; Yamashita & Kidera, 2001 ▸; Zhong *et al.*, 2002 ▸), it allows for the information related to charges to be extracted by electron two-dimensional (Mitsuoka *et al.*, 1999 ▸) and three-dimensional (3D) crystallography (Yonekura *et al.*, 2015 ▸; Yonekura & Maki-Yonekura, 2016 ▸) and single-particle cryo-EM (Yonekura & Maki-Yonekura, 2016 ▸). Also, ionic scattering factors could lead to a more accurate structure refinement against cryo-EM data (Yonekura & Maki-Yonekura, 2016 ▸). However, those studies assumed partial charges for a limited number of atoms including O and H of the side chains in titratable residues such as aspartate, glutamate, lysine, arginine and histidine.

The scattering factors of atoms and ions for X-rays and electrons were calculated (*e.g.* Doyle & Turner, 1968 ▸; Schmidt & Weiss, 1979 ▸; Rez *et al.*, 1994 ▸; Wang *et al.*, 1996 ▸; Su & Coppens, 1997 ▸; Macchi & Coppens, 2001 ▸) and parameterized previously (*e.g.* Doyle & Turner, 1968 ▸; Rez *et al.*, 1994 ▸; Waasmaier & Kirfel, 1995 ▸; Peng *et al.*, 1996 ▸; Su & Coppens, 1997 ▸; Peng, 1998 ▸; Macchi & Coppens, 2001 ▸; Colliex *et al.*, 2006 ▸), but there have been no reported scattering factors for chemically unstable ions involved in proteins and protein complexes except for O^−^. To fill the gap, we calculated the scattering factors of those ions by the multi-configuration Dirac–Fock (MCDF) method in this study. We only selected ions for the atoms, C, N, O, P and S that compose amino acids and nucleic acids, which are the major building blocks of biological molecules. The electron scattering factor of H^+^ is also presented. We then provide coefficients for the purpose of fitting the calculated scattering factors to the major curve models used in X-ray and electron crystallography and single-particle cryo-EM.

## 2. Calculations   

### 2.1. Calculation of radial wavefunctions   

Radial wavefunctions in the ground state were calculated for C, C^+^, C^−^, N, N^+^, N^−^, O, O^+^, O^−^, O^2−^, P, P^+^, P^−^, P^2+^, P^3+^, S, S^+^, S^−^, S^2+^, S^2−^, S^3+^ and S^4+^ by the MCDF method. The *GRASP2K* package (version 1.1, Jönsson *et al.*, 2013 ▸) was used to perform the extended optimal level calculation for self-consistent fields of wavefunctions. Isolated negative ions are known to be unstable with a short lifetime in vacuum and tend to suffer from the inherent lack of convergence by MCDF calculations. A practical approach to this problem is placing positive charges outside a given radius around the ion (Watson, 1958 ▸; Schmidt & Weiss, 1979 ▸; Rez *et al.*, 1994 ▸), but the resultant scattering curves differ significantly in the range sin*θ*/*λ* < 0.4 Å^−1^, depending on the size of the radius (Schmidt & Weiss, 1979 ▸). Previous studies (Wang *et al.*, 1996 ▸; Macchi & Coppens, 2001 ▸) showed that the Dirac–Fock calculation yielded good solutions for O^−^ and halides with no surrounding positive charges. Based on these studies, we carried out MCDF calculations in the absence of surrounding positive charges; this treatment was straightforward for O^−^, P^−^ and S^−^ as well as for the neutral atoms and positive ions with the exception of P^3+^ and S^4+^. For C^−^ and N^−^, starting from initial estimates of radial functions for the neutral atom and/or increasing array sizes in the *GRASP2K* programs yielded reasonable solutions. No stable solutions were obtained using this approach for the closed-shell ions: O^2−^, S^2−^, P^3+^ and S^4+^.

### 2.2. Conversion to scattering factors   

The scattering factors were calculated from relativistic wavefunctions as described in Su & Coppens (1997 ▸). Briefly, the radial charge density *ρ*(*r*) at a radius of *r* can be calculated as 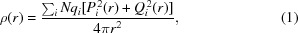
where *P_i_*(*r*) and *Q_i_*(*r*) represent the major and minor components, respectively, of the radial wavefunction for the *i*th orbital, and *Nq_i_* is the generalized occupation number for the corresponding orbital. The X-ray scattering factor *F_x_*(*s*) at *s* = sin*θ*/*λ* can then be converted from *ρ*(*r*) by Fourier transform in polar coordinates as 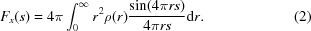



The integral in equation (2) was obtained by the composite-Simpson summation at a fine step of *r* given by cubic spline interpolation. The scattering factors were computed up to *s* = 12 Å^−1^. A python script called *scsumrhofft.py* (provided in the supporting information) was written for these calculations in equations (1) and (2).

### 2.3. Parameterization of scattering factors   

The *ScatCurve* package (Yonekura & Maki-Yonekura, 2016 ▸) was used to parameterize the X-ray scattering factors for the major curve models used in X-ray protein crystallography expressed in the form 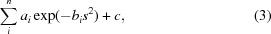
where *n* = 4 for the four Gaussians plus a constant model (Colliex *et al.*, 2006 ▸) and *n* = 5 for the five Gaussians plus a constant model (Waasmaier & Kirfel, 1995 ▸). The program *scatcurvefit* was modified to achieve a more robust minimization of the difference between the scattering factor and the model.

The X-ray scattering factor *F*_x_ was converted to the electron scattering factor *F*_el_ for an atom with *Z*_0_ electrons and *Z* nuclear charges using the Mott formula: 
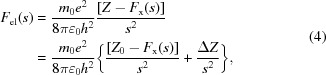
where *m*_0_ represents the electron mass, *e* is the electron charge, 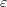
_0_ is the permittivity of free space, *h* is Planck’s constant, and (*m*_0_ *e* ^2^/8π∊_0_ *h* ^2^) = 0.02393366. Δ*Z* is the ionic charge and is defined as Δ*Z* = *Z* – *Z*_0_. The scattering factor of H^+^ was also obtained from equation (4) with *F*_x_ of H^+^ = 0 and Δ*Z* = 1. By using *scatcurvefit* (Yonekura & Maki-Yonekura, 2016 ▸) again, the electron scattering factors were param­eterized for the five Gaussians plus a charge term model (Peng, 1998 ▸) expressed as 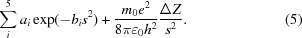



The calculated scattering factors and fitting to the curve model were evaluated from an *R*_scat_ factor (Peng, 1998 ▸; Yonekura & Maki-Yonekura, 2016 ▸) by using *scatcurvediff* and *scatcurvefit. R*_scat_ is defined as 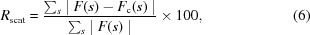
where *F* is the scattering amplitude computed in this work and *F*_c_ is the reference value or fitted value for the curve model.

## 3. Results and discussion   

### 3.1. Validation of scattering factors   

For validation of the X-ray scattering factors obtained from the MCDF calculations, we compared the calculated values for C, N, O, P, S and O^−^ with the reference values, which are tabulated in the *International Tables for Crystallography* (Colliex *et al.*, 2006 ▸). The tables contain these data up to *s* = 6 Å^−1^ for neutral atoms and 1.5 Å^−1^ for O^−^. *R*_scat_ values defined in equation (6) were calculated in these ranges (Table 1 ▸). The slight differences of 0.02–0.01% indicate that the values calculated in this study are consistent with the reference data. Plots of the calculated scattering factors and the reference curves appeared almost identical.

Relativistic effects are known to be small for light atoms (*e.g.* Wang *et al.*, 1996 ▸). Despite this, we included relativistic effects as most of the scattering factors in Colliex *et al.* (2006 ▸) adopted the relativistic calculation, even for the atoms concerned in this study. The MCDF calculation for those atoms does not require much computing cost on the current PC system. The reference scattering factor for O^−^ was non-relativistically computed (Colliex *et al.*, 2006 ▸) and the difference from our relativistic calculation is very small (Table 1 ▸), confirming a very small contribution from the relativistic term.

**Figure 3:**
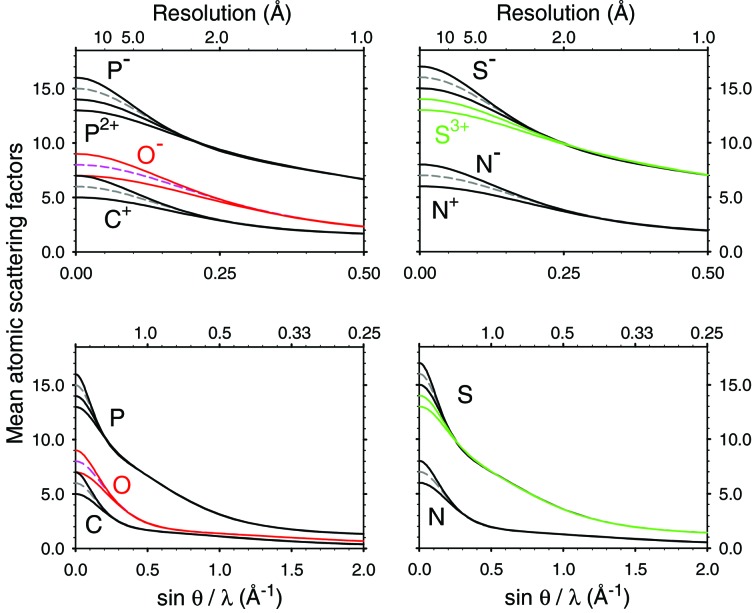
Plots of X-ray scattering factors obtained by our MCDF calculations. The X-ray scattering factors at sin*θ*/*λ* = 0 are equal to the number of electrons *Z*_0_. Dashed lines indicate plots of neutral atoms. Magenta and red curves correspond to the scattering factors of O, O^+^ and O^−^, and the green curves are those of S^2+^ and S^3+^. Plots in the lower row show the same curves as those in the upper row but up to sin *θ*/*λ* = 2 Å^−1^.

**Figure 4:**
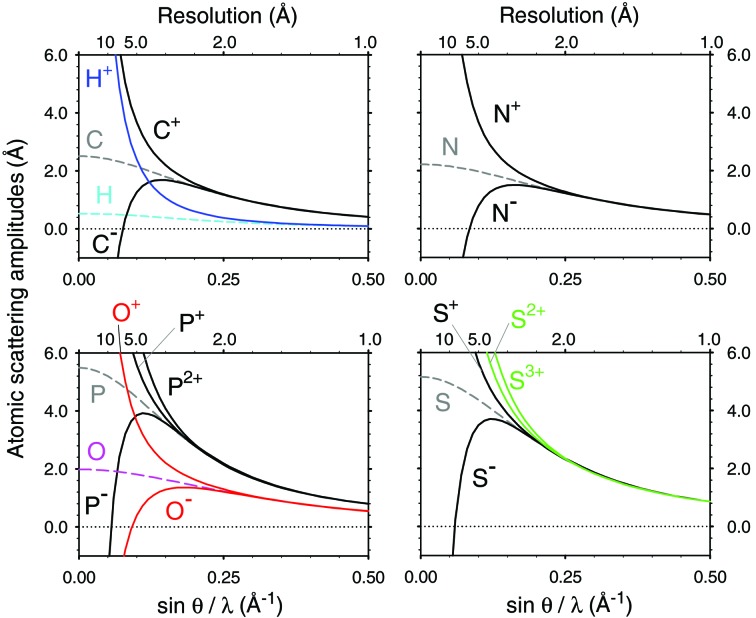
Plots of electron scattering factors converted from the X-ray scattering factors by the Mott formula (equation 4). Broken lines indicate plots for the neutral atoms. Negative ions show negative values in a lower sin *θ*/*λ* range. Magenta and red curves correspond to the scattering factors of O, O^+^ and O^−^, the cyan and blue curves are those of H, H^+^ and the green curves are those of S^2+^ and S^3+^. The scattering curve of H is derived from *International Tables for Crystallography* (Colliex *et al.*, 2006 ▸).

The scattering factors of the same atom with and without a charge match well when *s* = 0.2–0.3 Å^−1^ (Fig. 1 ▸). The curves of the calculated X-ray and electron scattering factors show these expected appearances, see Figs. 3 ▸ and 4 ▸, respectively. Figs. 3 ▸ and 4 ▸ also indicate that the point, where the curves of different charge states become overlapped, varies for each atom. In contrast, the electron scattering factors of H given in the *International Tables for Crystallography* (Colliex *et al.*, 2006 ▸) and of H^+^ derived in Hirai *et al.* (2007 ▸) do not match even beyond *s* = 0.5 Å^−1^ (see Fig. 1 of Yonekura & Maki-Yonekura, 2016 ▸). We calculated the electron scattering factor of H^+^ from equation (4) by simply setting* F*_x_ of H^+^ to 0 and Δ*Z* = 1. The new curve matches that of neutral H as sin*θ*/*λ* increases (Fig. 4 ▸). The calculated X-ray and electron scattering factors, including the new values for H^+^, are tabulated up to *s* = 6 Å^−1^ in Tables S1 and S2, respectively (see supporting information).

We also tried the standard quantum chemistry programs *Gaussian 09* (Frisch *et al.*, 2016 ▸) and *GAMESS* (Schmidt *et al.*, 1993 ▸) for the calculation of the scattering factors, with the hope of obtaining the scattering factors of O^2−^, S^2−^, P^3+^ and S^4+^, as the MCDF calculation gave no stable solution for these ions. Molecular orbital calculations with ROHF, UHF, MP2,3,4 and CCSD(T) were performed using the various Gaussian-type basis sets such as cc-pVTZ, cc-pV5Z, aug-cc-pVTZ, d-aug-cc-pVTZ and so on. Charge density *ρ* was then calculated on a sufficiently fine grid and converted to the radial scattering factor. The *R*_scat_ values, however, were around 0.5–1.0% against the references for neutral atoms and O^−^, and were much larger than the errors obtained from the MCDF calculations (Table 1 ▸), although the basis sets above were supposed to be available for quantitative calculation. Plots of the scattering factors appear to vary from the reference, particularly in the range of low sin*θ*/*λ*. This inconsistency may reflect that the Gaussian-type basis set cannot describe the cusps near the nucleus and inter-electron coalescent points (*e.g.* Pachucki & Komasa, 2004 ▸). Therefore, we did not adopt these calculations.

### 3.2. Parameterization of scattering factors   

We then parameterized the X-ray scattering factors in Table S1 for the four Gaussians plus a constant (Table 2 ▸) and five Gaussians plus a constant models (Table 3 ▸), and the electron scattering factors in Table S2 for the five Gaussians plus a charge term model (Table 4 ▸). Data up to *s* = 6 Å^−1^ were used for all X-ray scattering factors and data from *s* = 0.04 to 6 Å^−1^ for the electron scattering factors of the positive ions. Only data from *s* = 0.04 to 1.5 Å^−1^ were used for the electron scattering factors of the negative ions, as *R*_scat_ values between the scattering factors and the fitted curves were getting worse when including data beyond *s* = 1.5 Å^−1^. Calculated coefficients and *R*_scat_ values are summarized in Tables 2 ▸, 3 ▸ and 4 ▸. All the *R*_scat_ values are less than 0.09% and typically around 0.05–0.005%. The electron scattering factors of the negative ions give slightly worse *R*_scat_ values, but these values are comparable with those reported for other ions (Peng, 1998 ▸). Equations (4) and (5) are identical for H^+^ when all *a*_1–5_ = 0, and do not need to be parameterized for the five Gaussians plus a charge term model. Scattering factors of multivalent cations of phosphorus and sulfur may be necessary for some chemicals such as nicotinamide adenine dinucleotide phosphates [see the crystallographic information files (Brown & McMahon, 2002 ▸) in the *PHENIX* library (Adams *et al.*, 2010 ▸)]. Tables S1, S2 and 2–4 are provided in the supporting information for future reference.

### 3.3. Radiation damage   

Density losses in the negatively charged side chains of aspartate and glutamate were often observed in recent single-particle reconstructions (*e.g*. Bartesaghi *et al.*, 2014 ▸; Fromm *et al.*, 2015 ▸; Hryc *et al.*, 2017 ▸) and are likely to be interpreted as radiation damage (Fioravanti *et al.*, 2007 ▸) even when reconstructed from data sets collected with a low accumulated electron dose. Part of the density loss should be attributed to negatively charged atoms, and this can be checked if a map calculated without lower-resolution data recovers the corresponding density (Yonekura *et al.*, 2015 ▸). In contrast, an intense beam such as an X-ray-free electron laser causes ionization damage to samples and this may affect the diffraction patterns (Hau-Riege, 2007 ▸). The scattering factors of these radicals losing some electrons may help us to treat the data properly (Hau-Riege, 2007 ▸).

## 4. Conclusions   

We have provided the X-ray and electron scattering factors of C^+^, C^−^, N^+^, N^−^, O^+^, O^−^, P^+^, P^−^, P^2+^, S^+^, S^−^, S^2+^ and S^3+^, the electron scattering factor of H^+^ and the coefficients for the four Gaussians plus a constant, the five Gaussians plus a constant and the five Gaussians plus a charge term models. The scattering factors of partially charged atoms and the fitting parameters for a curve model can be obtained by a linear combination of those for neutral and fully ionized atoms using *scatcurvecomb* (as in Yonekura & Maki-Yonekura, 2016 ▸). We are now testing the calculated values in this study for structure refinement against data obtained by electron 3D crystallography and single-particle cryo-EM.

## Supplementary Material

Supplementary Tables. DOI: 10.1107/S2052252518005237/fq5001sup1.pdf

Supplementary code.. DOI: 10.1107/S2052252518005237/fq5001sup2.zip

## Figures and Tables

**Table 1:** *R*_scat_ factors of X-ray scattering factors against the reference curves

Atom	*R*_scat_ † (%)
C	0.0125
N	0.0194
O	0.0223
O^−^	0.0223
P	0.0047
S	0.0075

† Defined in equation (6). Calculated between the scattering factors and the reference values in *International Tables for Crystallography* (Colliex *et al.*, 2006 ▸). Summation over data from sin*θ*/*λ* = 0 to 1.5 Å^−1^ for O^−^ and from 0 to 6 Å^−1^ for all the others.

**Table 2:** Parameterization of X-ray scattering factors of ions for the four Gaussians plus a constant model

	*a*_1_	*a*_2_	*a*_3_	*a*_4_	*c*	*R*_scat_ †
Ion	*b*_1_	*b*_2_	*b*_3_	*b*_4_		(%)
C^+^	6.727E+0	1.439E+0	2.191E+0	9.913E-1	−6.349E+0	0.0154
	4.479E-3	6.231E-1	1.313E+1	2.971E+1		
C^−^	1.598E+0	2.161E+0	2.360E+0	6.549E-1	2.238E-1	0.0606
	5.843E-1	1.346E+1	3.831E+1	1.405E+2		
N^+^	1.570E+0	2.068E+0	1.847E+0	2.870E-1	2.271E-1	0.0025
	4.122E-1	8.064E+0	1.665E+1	3.340E+1		
N^−^	1.569E+0	2.640E+0	2.795E+0	7.386E-1	2.551E-1	0.0394
	4.393E-1	9.202E+0	2.624E+1	9.062E+1		
O^+^	1.553E+0	2.237E+0	2.466E+0	5.079E-1	2.367E-1	0.0015
	3.148E-1	5.618E+0	1.191E+1	2.504E+1		
O^−^	1.535E+0	3.038E+0	3.280E+0	8.555E-1	2.886E-1	0.0346
	3.459E-1	6.556E+0	1.876E+1	6.328E+1		
P^+^	1.732E+0	6.472E+0	3.698E+0	9.940E-1	1.105E+0	0.0065
	5.089E-1	1.893E+0	2.424E+1	5.215E+1		
P^−^	1.990E+0	6.243E+0	4.686E+0	1.928E+0	1.146E+0	0.0423
	5.903E-1	1.967E+0	3.046E+1	1.031E+2		
P^2+^	1.825E+0	6.368E+0	3.076E+0	6.076E-1	1.124E+0	0.0046
	5.431E-1	1.924E+0	2.114E+1	4.185E+1		
S^+^	1.419E+0	6.923E+0	4.736E+0	1.076E+0	8.458E-1	0.0115
	2.405E-1	1.459E+0	2.028E+1	4.519E+1		
S^−^	1.505E+0	6.721E+0	5.614E+0	2.130E+0	1.024E+0	0.0372
	3.506E-1	1.507E+0	2.411E+1	7.728E+1		
S^2+^	1.442E+0	6.787E+0	4.206E+0	5.939E-1	9.715E-1	0.0092
	3.088E-1	1.488E+0	1.846E+1	3.950E+1		
S^3+^	1.611E+0	6.550E+0	3.434E+0	3.284E-1	1.078E+0	0.0051
	4.062E-1	1.543E+0	1.637E+1	3.422E+1		

† Defined in equation (6). Calculated between the scattering factors and the fitted curves. Summation over data from sin*θ*/*λ* = 0 to 2 Å^−1^.

**Table 3:** Parameterization of X-ray scattering factors of ions for the five Gaussians plus a constant model

	*a*_1_	*a*_2_	*a*_3_	*a*_4_	*a*_5_	*c*	*R*_scat_ †
Ion	*b*_1_	*b*_2_	*b*_3_	*b*_4_	*b*_5_		(%)
C^+^	8.431E-2	5.957E-1	1.145E+0	2.224E+0	9.528E-1	−2.457E-3	0.0194
	4.787E-2	2.273E-1	7.042E-1	1.328E+1	3.012E+1		
C^−^	5.946E-1	1.229E+0	2.295E+0	2.257E+0	6.050E-1	1.642E-2	0.0516
	1.840E-1	7.149E-1	1.408E+1	4.041E+1	1.463E+2		
N^+^	1.670E-1	6.515E-1	9.987E-1	2.749E+0	1.427E+0	5.943E-3	0.0228
	6.426E-2	2.094E-1	5.482E-1	9.105E+0	2.215E+1		
N^−^	6.423E-1	1.169E+0	2.685E+0	2.758E+0	7.215E-1	2.224E-2	0.0403
	1.451E-1	5.304E-1	9.338E+0	2.664E+1	9.161E+1		
O^+^	6.042E-1	1.171E+0	2.398E+0	2.376E+0	4.253E-1	2.527E-2	0.0047
	1.078E-1	3.764E-1	5.797E+0	1.247E+1	2.613E+1		
O^−^	6.950E-1	1.102E+0	3.023E+0	3.287E+0	8.611E-1	2.919E-2	0.0367
	1.195E-1	4.139E-1	6.546E+0	1.869E+1	6.309E+1		
P^+^	1.496E+0	1.992E+0	5.676E+0	3.739E+0	9.422E-1	1.546E-1	0.0107
	7.120E-2	9.188E-1	1.987E+0	2.446E+1	5.304E+1		
P^−^	1.505E+0	2.539E+0	5.185E+0	4.696E+0	1.909E+0	1.595E-1	0.0431
	7.246E-2	1.008E+0	2.097E+0	3.061E+1	1.036E+2		
P^2+^	1.501E+0	2.253E+0	5.421E+0	3.145E+0	5.236E-1	1.563E-1	0.0084
	7.155E-2	9.661E-1	2.041E+0	2.148E+1	4.363E+1		
S^+^	1.477E+0	1.268E+0	6.299E+0	4.788E+0	1.011E+0	1.569E-1	0.0155
	6.175E-2	6.681E-1	1.521E+0	2.047E+1	4.613E+1		
S^−^	1.483E+0	1.597E+0	6.016E+0	5.623E+0	2.114E+0	1.610E-1	0.0397
	6.259E-2	7.320E-1	1.570E+0	2.419E+1	7.758E+1		
S^2+^	1.483E+0	1.520E+0	6.052E+0	4.257E+0	5.300E-1	1.594E-1	0.0131
	6.220E-2	7.229E-1	1.558E+0	1.865E+1	4.092E+1		
S^3+^	1.489E+0	1.983E+0	5.618E+0	3.476E+0	2.716E-1	1.626E-1	0.0084
	6.277E-2	8.002E-1	1.631E+0	1.656E+1	3.620E+1		

† Defined in equation (6). Calculated between the scattering factors and the fitted curves. Summation over data from sin*θ*/*λ *= 0 to 6 Å^−1^.

**Table 4:** Parameterization of electron scattering factors of ions for the five Gaussians plus a charge term model

	*a*_1_	*a*_2_	*a*_3_	*a*_4_	*a*_5_	*R*_scat_ †
Ion	*b*_1_	*b*_2_	*b*_3_	*b*_4_	*b*_5_	(%)
C^+^	2.079E-2	9.266E-2	2.949E-1	6.812E-1	3.304E-1	0.0156
	5.950E-2	5.359E-1	2.760E+0	9.283E+0	2.442E+1	
C^-^	2.248E-1	8.254E-1	1.769E+0	1.690E+0	6.994E-1	0.0810
	5.518E-1	4.308E+0	1.600E+1	5.196E+1	1.708E+2	
N^+^	2.296E-2	1.004E-1	3.289E-1	6.546E-1	2.733E-1	0.0132
	5.522E-2	4.910E-1	2.402E+0	7.751E+0	2.051E+1	
N^−^	2.192E-1	7.256E-1	1.398E+0	1.245E+0	4.381E-1	0.0430
	4.784E-1	3.389E+0	1.171E+1	3.604E+1	1.125E+2	
O^+^	2.439E-2	1.036E-1	3.360E-1	6.112E-1	2.447E-1	0.0120
	5.082E-2	4.390E-1	2.036E+0	6.407E+0	1.710E+1	
O^−^	2.236E-1	6.923E-1	1.176E+0	9.354E-1	2.821E-1	0.0218
	4.372E-1	2.918E+0	9.670E+0	2.868E+1	8.489E+1	
P^+^	6.191E-2	3.154E-1	6.324E-1	1.661E+0	1.027E+0	0.0447
	6.525E-2	6.224E-1	3.009E+0	1.258E+1	3.411E+1	
P^−^	4.590E-1	1.002E+0	2.724E+0	3.228E+0	1.336E+0	0.0728
	5.019E-1	3.545E+0	1.445E+1	4.313E+1	1.303E+2	
P^2+^	4.997E-2	2.497E-1	4.690E-1	1.099E+0	6.144E-1	0.0233
	5.712E-2	5.372E-1	2.398E+0	1.051E+1	2.704E+1	
S^+^	6.232E-2	3.129E-1	6.541E-1	1.742E+0	9.377E-1	0.0391
	6.149E-2	5.785E-1	2.848E+0	1.107E+1	2.978E+1	
S^−^	4.496E-1	9.810E-1	2.598E+0	2.717E+0	8.614E-1	0.0543
	4.656E-1	3.259E+0	1.233E+1	3.583E+1	1.055E+2	
S^2+^	5.029E-2	2.440E-1	4.671E-1	1.196E+0	7.100E-1	0.0230
	5.364E-2	4.908E-1	2.186E+0	8.911E+0	2.266E+1	
S^3+^	3.991E-2	1.787E-1	3.465E-1	7.425E-1	5.600E-1	0.0124
	4.652E-2	4.001E-1	1.626E+0	6.936E+0	1.724E+1	

† Defined in equation (6). Calculated between the scattering factors and the fitted curves. Summation over data from sin*θ*/*λ* = 0.04 to 6 Å^−1^ for the positive ions and from 0.04 to 1.5 Å^−1^ for the negative ions.
