# Supplementary material for: Ionic scattering factors of atoms that compose biological molecules
Source: IUCrJ. 2018 Apr 27;5(Pt 3):348–53. doi: 10.1107/S2052252518005237 (PMC5929380; doi:10.1107/S2052252518005237)
Supplement: Supplementary file 1 [file m-05-00348-sup1.pdf]

IUCrJ

Volume 5 (2018)

Supporting information for article:

Ionic scattering factors of atoms that compose biological molecules

Koji Yonekura, Rei Matsuoka, Yoshiki Yamashita, Tsutomu Yamane, Mitsunori Ikeguchi, Akinori Kidera and Saori Maki-Yonekura

Supporting Information

Ionic scattering factors of atoms that compose biological molecules

Koji Yonekura*, Rei Matsuoka, Yoshiki Yamashita, Tsutomu Yamane,

Mitsunori Ikeguchi, Akinori Kidera, and Saori Maki-Yonekura

* To whom correspondence should be addressed.

E-mail: yone@spring8.or.jp

Table S1.

X-ray scattering factors of ions

sinθ/ λ (Å^{-1})	C^+	C^-	N^+	N^-	O^+	O^-
0.00	5.000e+0	7.000e+0	6.000e+0	8.000e+0	7.000e+0	9.000e+0
0.01	4.994e+0	6.978e+0	5.994e+0	7.983e+0	6.995e+0	8.986e+0
0.02	4.976e+0	6.914e+0	5.977e+0	7.933e+0	6.978e+0	8.945e+0
0.03	4.947e+0	6.812e+0	5.949e+0	7.852e+0	6.951e+0	8.878e+0
0.04	4.907e+0	6.675e+0	5.909e+0	7.742e+0	6.913e+0	8.785e+0
0.05	4.856e+0	6.511e+0	5.859e+0	7.607e+0	6.864e+0	8.670e+0
0.06	4.794e+0	6.325e+0	5.798e+0	7.449e+0	6.806e+0	8.534e+0
0.07	4.724e+0	6.124e+0	5.729e+0	7.274e+0	6.738e+0	8.381e+0
0.08	4.645e+0	5.912e+0	5.650e+0	7.084e+0	6.662e+0	8.211e+0
0.09	4.559e+0	5.694e+0	5.563e+0	6.883e+0	6.577e+0	8.029e+0
0.10	4.465e+0	5.473e+0	5.468e+0	6.674e+0	6.484e+0	7.836e+0
0.11	4.367e+0	5.253e+0	5.367e+0	6.460e+0	6.383e+0	7.636e+0
0.12	4.263e+0	5.037e+0	5.261e+0	6.244e+0	6.277e+0	7.429e+0
0.13	4.156e+0	4.824e+0	5.149e+0	6.028e+0	6.164e+0	7.218e+0
0.14	4.047e+0	4.619e+0	5.033e+0	5.813e+0	6.047e+0	7.006e+0
0.15	3.935e+0	4.420e+0	4.913e+0	5.601e+0	5.925e+0	6.793e+0
0.16	3.823e+0	4.230e+0	4.792e+0	5.393e+0	5.800e+0	6.580e+0
0.17	3.710e+0	4.049e+0	4.668e+0	5.191e+0	5.671e+0	6.369e+0
0.18	3.598e+0	3.876e+0	4.544e+0	4.994e+0	5.540e+0	6.161e+0
0.19	3.488e+0	3.712e+0	4.419e+0	4.803e+0	5.408e+0	5.957e+0
0.20	3.379e+0	3.558e+0	4.294e+0	4.620e+0	5.275e+0	5.757e+0
0.22	3.169e+0	3.275e+0	4.048e+0	4.274e+0	5.007e+0	5.372e+0
0.24	2.973e+0	3.026e+0	3.810e+0	3.958e+0	4.742e+0	5.010e+0
0.25	2.880e+0	2.914e+0	3.695e+0	3.811e+0	4.612e+0	4.838e+0
0.26	2.791e+0	2.810e+0	3.583e+0	3.671e+0	4.483e+0	4.672e+0
0.28	2.625e+0	2.622e+0	3.368e+0	3.413e+0	4.232e+0	4.358e+0
0.30	2.476e+0	2.459e+0	3.168e+0	3.182e+0	3.993e+0	4.070e+0
0.32	2.343e+0	2.320e+0	2.983e+0	2.976e+0	3.766e+0	3.806e+0
0.34	2.225e+0	2.200e+0	2.814e+0	2.794e+0	3.553e+0	3.565e+0
0.35	2.172e+0	2.147e+0	2.736e+0	2.710e+0	3.452e+0	3.453e+0
0.36	2.122e+0	2.097e+0	2.660e+0	2.632e+0	3.354e+0	3.347e+0
0.38	2.031e+0	2.009e+0	2.522e+0	2.490e+0	3.170e+0	3.149e+0
0.40	1.952e+0	1.934e+0	2.397e+0	2.364e+0	3.000e+0	2.970e+0
0.42	1.884e+0	1.869e+0	2.285e+0	2.253e+0	2.844e+0	2.809e+0
0.44	1.824e+0	1.812e+0	2.185e+0	2.156e+0	2.701e+0	2.665e+0
0.45	1.797e+0	1.787e+0	2.140e+0	2.112e+0	2.635e+0	2.598e+0
0.46	1.771e+0	1.763e+0	2.097e+0	2.071e+0	2.572e+0	2.535e+0
0.48	1.726e+0	1.720e+0	2.018e+0	1.996e+0	2.454e+0	2.419e+0
0.50	1.685e+0	1.682e+0	1.948e+0	1.929e+0	2.348e+0	2.314e+0
0.55	1.602e+0	1.603e+0	1.806e+0	1.795e+0	2.125e+0	2.099e+0

0.60	1.536e+0	1.538e+0	1.699e+0	1.694e+0	1.953e+0	1.935e+0
0.65	1.479e+0	1.480e+0	1.617e+0	1.615e+0	1.820e+0	1.809e+0
0.70	1.426e+0	1.426e+0	1.551e+0	1.551e+0	1.717e+0	1.711e+0
0.80	1.325e+0	1.322e+0	1.445e+0	1.446e+0	1.569e+0	1.568e+0
0.90	1.224e+0	1.217e+0	1.355e+0	1.353e+0	1.464e+0	1.464e+0
1.00	1.121e+0	1.112e+0	1.268e+0	1.264e+0	1.378e+0	1.377e+0
1.10	1.019e+0	1.009e+0	1.181e+0	1.175e+0	1.300e+0	1.298e+0
1.20	9.206e-1	9.110e-1	1.095e+0	1.088e+0	1.224e+0	1.220e+0
1.30	8.282e-1	8.189e-1	1.009e+0	1.002e+0	1.149e+0	1.144e+0
1.40	7.425e-1	7.338e-1	9.268e-1	9.190e-1	1.075e+0	1.069e+0
1.50	6.642e-1	6.561e-1	8.480e-1	8.403e-1	1.001e+0	9.950e-1
1.60	5.933e-1	5.859e-1	7.738e-1	7.664e-1	9.302e-1	9.237e-1
1.70	5.295e-1	5.229e-1	7.047e-1	6.977e-1	8.618e-1	8.552e-1
1.80	4.725e-1	4.666e-1	6.408e-1	6.342e-1	7.966e-1	7.902e-1
1.90	4.217e-1	4.164e-1	5.821e-1	5.760e-1	7.350e-1	7.289e-1
2.00	3.767e-1	3.719e-1	5.285e-1	5.229e-1	6.773e-1	6.715e-1
2.50	2.176e-1	2.149e-1	3.266e-1	3.232e-1	4.457e-1	4.417e-1
3.00	1.306e-1	1.290e-1	2.056e-1	2.035e-1	2.941e-1	2.914e-1
3.50	8.158e-2	8.062e-2	1.331e-1	1.318e-1	1.972e-1	1.955e-1
4.00	5.298e-2	5.236e-2	8.877e-2	8.789e-2	1.351e-1	1.339e-1
5.00	2.470e-2	2.442e-2	4.291e-2	4.249e-2	6.778e-2	6.721e-2
6.00	1.285e-2	1.270e-2	2.282e-2	2.260e-2	3.691e-2	3.660e-2

$\sin\theta/\lambda$ (Å^{-1})	P^{+}	P^{-}	P^{2+}	S^{+}	S^{-}	S^{2+}	S^{3+}
0.00	1.400e+1	1.600e+1	1.300e+1	1.500e+1	1.700e+1	1.400e+1	1.300e+1
0.01	1.398e+1	1.596e+1	1.299e+1	1.498e+1	1.697e+1	1.399e+1	1.299e+1
0.02	1.394e+1	1.586e+1	1.296e+1	1.494e+1	1.687e+1	1.396e+1	1.297e+1
0.03	1.386e+1	1.568e+1	1.291e+1	1.486e+1	1.672e+1	1.390e+1	1.293e+1
0.04	1.376e+1	1.545e+1	1.284e+1	1.476e+1	1.651e+1	1.382e+1	1.288e+1
0.05	1.363e+1	1.517e+1	1.275e+1	1.462e+1	1.626e+1	1.373e+1	1.281e+1
0.06	1.347e+1	1.485e+1	1.264e+1	1.447e+1	1.596e+1	1.361e+1	1.273e+1
0.07	1.330e+1	1.451e+1	1.252e+1	1.429e+1	1.564e+1	1.348e+1	1.263e+1
0.08	1.310e+1	1.414e+1	1.238e+1	1.409e+1	1.529e+1	1.333e+1	1.253e+1
0.09	1.289e+1	1.377e+1	1.223e+1	1.387e+1	1.492e+1	1.317e+1	1.241e+1
0.10	1.267e+1	1.339e+1	1.207e+1	1.364e+1	1.454e+1	1.299e+1	1.228e+1
0.11	1.244e+1	1.302e+1	1.191e+1	1.339e+1	1.415e+1	1.281e+1	1.214e+1
0.12	1.220e+1	1.265e+1	1.173e+1	1.314e+1	1.377e+1	1.261e+1	1.200e+1
0.13	1.196e+1	1.230e+1	1.155e+1	1.288e+1	1.339e+1	1.241e+1	1.185e+1
0.14	1.171e+1	1.196e+1	1.136e+1	1.261e+1	1.302e+1	1.220e+1	1.169e+1
0.15	1.147e+1	1.164e+1	1.117e+1	1.235e+1	1.266e+1	1.199e+1	1.153e+1
0.16	1.123e+1	1.133e+1	1.098e+1	1.208e+1	1.231e+1	1.177e+1	1.136e+1
0.17	1.099e+1	1.104e+1	1.079e+1	1.182e+1	1.198e+1	1.156e+1	1.120e+1
0.18	1.076e+1	1.077e+1	1.061e+1	1.156e+1	1.166e+1	1.134e+1	1.103e+1
0.19	1.054e+1	1.051e+1	1.042e+1	1.131e+1	1.136e+1	1.113e+1	1.086e+1
0.20	1.033e+1	1.027e+1	1.024e+1	1.106e+1	1.107e+1	1.092e+1	1.069e+1
0.22	9.923e+0	9.836e+0	9.889e+0	1.060e+1	1.054e+1	1.052e+1	1.035e+1
0.24	9.554e+0	9.457e+0	9.557e+0	1.016e+1	1.008e+1	1.013e+1	1.003e+1
0.25	9.383e+0	9.286e+0	9.399e+0	9.961e+0	9.869e+0	9.947e+0	9.869e+0
0.26	9.220e+0	9.125e+0	9.247e+0	9.768e+0	9.671e+0	9.770e+0	9.714e+0
0.28	8.918e+0	8.833e+0	8.959e+0	9.411e+0	9.313e+0	9.435e+0	9.415e+0
0.30	8.644e+0	8.573e+0	8.690e+0	9.088e+0	8.997e+0	9.126e+0	9.134e+0
0.32	8.394e+0	8.338e+0	8.441e+0	8.798e+0	8.717e+0	8.843e+0	8.868e+0
0.34	8.164e+0	8.122e+0	8.208e+0	8.536e+0	8.468e+0	8.582e+0	8.619e+0
0.35	8.056e+0	8.020e+0	8.097e+0	8.414e+0	8.353e+0	8.460e+0	8.500e+0
0.36	7.951e+0	7.921e+0	7.989e+0	8.298e+0	8.243e+0	8.342e+0	8.385e+0
0.38	7.750e+0	7.730e+0	7.781e+0	8.080e+0	8.038e+0	8.120e+0	8.164e+0
0.40	7.558e+0	7.547e+0	7.583e+0	7.878e+0	7.848e+0	7.913e+0	7.956e+0
0.42	7.373e+0	7.370e+0	7.391e+0	7.690e+0	7.670e+0	7.719e+0	7.758e+0
0.44	7.193e+0	7.195e+0	7.206e+0	7.512e+0	7.500e+0	7.535e+0	7.570e+0
0.45	7.104e+0	7.109e+0	7.114e+0	7.426e+0	7.418e+0	7.447e+0	7.479e+0
0.46	7.016e+0	7.022e+0	7.024e+0	7.342e+0	7.337e+0	7.360e+0	7.389e+0
0.48	6.842e+0	6.851e+0	6.846e+0	7.178e+0	7.178e+0	7.190e+0	7.214e+0
0.50	6.670e+0	6.680e+0	6.669e+0	7.018e+0	7.022e+0	7.026e+0	7.043e+0
0.55	6.243e+0	6.255e+0	6.237e+0	6.629e+0	6.639e+0	6.628e+0	6.633e+0
0.60	5.822e+0	5.834e+0	5.813e+0	6.247e+0	6.259e+0	6.241e+0	6.238e+0
0.65	5.411e+0	5.420e+0	5.401e+0	5.870e+0	5.882e+0	5.861e+0	5.852e+0
0.70	5.014e+0	5.021e+0	5.004e+0	5.498e+0	5.508e+0	5.488e+0	5.476e+0
0.80	4.280e+0	4.284e+0	4.271e+0	4.784e+0	4.791e+0	4.775e+0	4.761e+0

0.90	3.646e+0	3.648e+0	3.639e+0	4.134e+0	4.138e+0	4.126e+0	4.113e+0
1.00	3.120e+0	3.122e+0	3.114e+0	3.567e+0	3.570e+0	3.560e+0	3.550e+0
1.10	2.696e+0	2.698e+0	2.692e+0	3.089e+0	3.091e+0	3.084e+0	3.075e+0
1.20	2.362e+0	2.364e+0	2.359e+0	2.697e+0	2.699e+0	2.693e+0	2.686e+0
1.30	2.102e+0	2.104e+0	2.100e+0	2.382e+0	2.383e+0	2.378e+0	2.373e+0
1.40	1.901e+0	1.903e+0	1.899e+0	2.131e+0	2.133e+0	2.128e+0	2.124e+0
1.50	1.746e+0	1.747e+0	1.744e+0	1.933e+0	1.935e+0	1.931e+0	1.928e+0
1.60	1.625e+0	1.626e+0	1.624e+0	1.778e+0	1.779e+0	1.776e+0	1.773e+0
1.70	1.530e+0	1.530e+0	1.529e+0	1.654e+0	1.656e+0	1.653e+0	1.651e+0
1.80	1.452e+0	1.453e+0	1.452e+0	1.556e+0	1.557e+0	1.555e+0	1.554e+0
1.90	1.388e+0	1.389e+0	1.388e+0	1.476e+0	1.477e+0	1.476e+0	1.475e+0
2.00	1.333e+0	1.334e+0	1.333e+0	1.410e+0	1.411e+0	1.410e+0	1.409e+0
2.50	1.123e+0	1.122e+0	1.123e+0	1.183e+0	1.182e+0	1.183e+0	1.184e+0
3.00	9.429e-1	9.420e-1	9.437e-1	1.010e+0	1.009e+0	1.010e+0	1.011e+0
3.50	7.777e-1	7.769e-1	7.785e-1	8.500e-1	8.491e-1	8.507e-1	8.517e-1
4.00	6.326e-1	6.319e-1	6.332e-1	7.052e-1	7.044e-1	7.059e-1	7.067e-1
5.00	4.112e-1	4.107e-1	4.116e-1	4.738e-1	4.733e-1	4.743e-1	4.748e-1
6.00	2.678e-1	2.675e-1	2.681e-1	3.165e-1	3.161e-1	3.168e-1	3.171e-1

Table S2.

Electron scattering factors of ions

$\sin\theta/\lambda$ (Å^{-1})	H^+	C^+	C^-	N^+	N^-	O^+	O^-
0.04	1.496e+1	1.635e+1	-1.010e+1	1.632e+1	-1.110e+1	1.626e+1	-1.175e+1
0.05	9.573e+0	1.096e+1	-4.893e+0	1.093e+1	-5.809e+0	1.087e+1	-6.416e+0
0.06	6.648e+0	8.015e+0	-2.163e+0	7.988e+0	-2.988e+0	7.937e+0	-3.553e+0
0.07	4.884e+0	6.233e+0	-6.052e-1	6.210e+0	-1.337e+0	6.162e+0	-1.859e+0
0.08	3.740e+0	5.067e+0	3.298e-1	5.049e+0	-3.130e-1	5.005e+0	-7.902e-1
0.09	2.955e+0	4.259e+0	9.050e-1	4.247e+0	3.466e-1	4.206e+0	-8.569e-2
0.10	2.393e+0	3.673e+0	1.261e+0	3.666e+0	7.802e-1	3.630e+0	3.916e-1
0.11	1.978e+0	3.231e+0	1.477e+0	3.229e+0	1.067e+0	3.198e+0	7.208e-1
0.12	1.662e+0	2.886e+0	1.601e+0	2.891e+0	1.256e+0	2.864e+0	9.489e-1
0.13	1.416e+0	2.611e+0	1.665e+0	2.622e+0	1.377e+0	2.600e+0	1.107e+0
0.14	1.221e+0	2.385e+0	1.687e+0	2.402e+0	1.449e+0	2.385e+0	1.214e+0
0.15	1.064e+0	2.197e+0	1.680e+0	2.220e+0	1.488e+0	2.207e+0	1.284e+0
0.16	9.349e-1	2.036e+0	1.655e+0	2.065e+0	1.502e+0	2.057e+0	1.328e+0
0.17	8.282e-1	1.896e+0	1.616e+0	1.931e+0	1.498e+0	1.929e+0	1.351e+0
0.18	7.387e-1	1.774e+0	1.569e+0	1.815e+0	1.482e+0	1.817e+0	1.358e+0
0.19	6.630e-1	1.666e+0	1.517e+0	1.711e+0	1.456e+0	1.718e+0	1.354e+0
0.20	5.983e-1	1.568e+0	1.461e+0	1.619e+0	1.424e+0	1.631e+0	1.342e+0
0.22	4.945e-1	1.400e+0	1.348e+0	1.460e+0	1.348e+0	1.480e+0	1.299e+0
0.24	4.155e-1	1.258e+0	1.236e+0	1.325e+0	1.264e+0	1.354e+0	1.242e+0
0.25	3.829e-1	1.195e+0	1.182e+0	1.266e+0	1.221e+0	1.298e+0	1.211e+0
0.26	3.540e-1	1.136e+0	1.130e+0	1.210e+0	1.178e+0	1.245e+0	1.178e+0
0.28	3.053e-1	1.030e+0	1.031e+0	1.109e+0	1.095e+0	1.150e+0	1.112e+0
0.30	2.659e-1	9.371e-1	9.416e-1	1.019e+0	1.015e+0	1.066e+0	1.045e+0
0.32	2.337e-1	8.547e-1	8.602e-1	9.388e-1	9.404e-1	9.896e-1	9.802e-1
0.34	2.070e-1	7.815e-1	7.867e-1	8.666e-1	8.709e-1	9.207e-1	9.181e-1
0.35	1.954e-1	7.479e-1	7.528e-1	8.332e-1	8.381e-1	8.886e-1	8.883e-1
0.36	1.847e-1	7.162e-1	7.207e-1	8.014e-1	8.066e-1	8.580e-1	8.593e-1
0.38	1.657e-1	6.578e-1	6.614e-1	7.423e-1	7.476e-1	8.006e-1	8.040e-1
0.40	1.496e-1	6.055e-1	6.082e-1	6.886e-1	6.935e-1	7.480e-1	7.524e-1
0.42	1.357e-1	5.585e-1	5.605e-1	6.398e-1	6.440e-1	6.996e-1	7.042e-1
0.44	1.236e-1	5.163e-1	5.177e-1	5.952e-1	5.988e-1	6.550e-1	6.595e-1
0.45	1.182e-1	4.968e-1	4.979e-1	5.745e-1	5.777e-1	6.341e-1	6.384e-1
0.46	1.131e-1	4.783e-1	4.792e-1	5.546e-1	5.575e-1	6.140e-1	6.181e-1
0.48	1.039e-1	4.440e-1	4.446e-1	5.175e-1	5.199e-1	5.761e-1	5.798e-1
0.50	9.573e-2	4.131e-1	4.134e-1	4.836e-1	4.855e-1	5.411e-1	5.443e-1
0.55	7.912e-2	3.480e-1	3.479e-1	4.110e-1	4.118e-1	4.649e-1	4.669e-1
0.60	6.648e-2	2.968e-1	2.967e-1	3.524e-1	3.528e-1	4.020e-1	4.032e-1
0.65	5.665e-2	2.561e-1	2.560e-1	3.049e-1	3.050e-1	3.501e-1	3.507e-1
0.70	4.884e-2	2.234e-1	2.234e-1	2.662e-1	2.662e-1	3.069e-1	3.072e-1
0.80	3.740e-2	1.748e-1	1.749e-1	2.077e-1	2.077e-1	2.405e-1	2.405e-1

0.90	2.955e-2	1.411e-1	1.413e-1	1.668e-1	1.669e-1	1.931e-1	1.931e-1
1.00	2.393e-2	1.168e-1	1.170e-1	1.372e-1	1.373e-1	1.585e-1	1.585e-1
1.10	1.978e-2	9.853e-2	9.872e-2	1.151e-1	1.152e-1	1.325e-1	1.326e-1
1.20	1.662e-2	8.442e-2	8.458e-2	9.815e-2	9.827e-2	1.126e-1	1.127e-1
1.30	1.416e-2	7.324e-2	7.337e-2	8.484e-2	8.495e-2	9.702e-2	9.710e-2
1.40	1.221e-2	6.420e-2	6.431e-2	7.416e-2	7.426e-2	8.457e-2	8.464e-2
1.50	1.064e-2	5.676e-2	5.684e-2	6.544e-2	6.552e-2	7.445e-2	7.451e-2
1.60	9.349e-3	5.055e-2	5.062e-2	5.821e-2	5.828e-2	6.610e-2	6.616e-2
1.70	8.282e-3	4.530e-2	4.536e-2	5.214e-2	5.219e-2	5.912e-2	5.917e-2
1.80	7.387e-3	4.083e-2	4.088e-2	4.698e-2	4.702e-2	5.321e-2	5.326e-2
1.90	6.630e-3	3.698e-2	3.702e-2	4.255e-2	4.259e-2	4.817e-2	4.821e-2
2.00	5.983e-3	3.365e-2	3.368e-2	3.872e-2	3.876e-2	4.381e-2	4.385e-2
2.50	3.829e-3	2.214e-2	2.215e-2	2.556e-2	2.557e-2	2.893e-2	2.894e-2
3.00	2.659e-3	1.561e-2	1.561e-2	1.807e-2	1.807e-2	2.049e-2	2.050e-2
3.50	1.954e-3	1.156e-2	1.157e-2	1.342e-2	1.342e-2	1.524e-2	1.525e-2
4.00	1.496e-3	8.896e-3	8.897e-3	1.034e-2	1.034e-2	1.176e-2	1.177e-2
5.00	9.573e-4	5.720e-3	5.721e-3	6.660e-3	6.661e-3	7.594e-3	7.594e-3
6.00	6.648e-4	3.980e-3	3.980e-3	4.639e-3	4.639e-3	5.294e-3	5.294e-3

$\sin\theta/\lambda$ (Å^{-1})	P^{+}	P^{-}	P^{2+}	S^{+}	S^{-}	S^{2+}	S^{3+}
0.04	1.856e+1	-6.746e+0	3.236e+1	1.859e+1	-7.689e+0	3.253e+1	4.672e+1
0.05	1.313e+1	-1.627e+0	2.156e+1	1.316e+1	-2.489e+0	2.175e+1	3.055e+1
0.06	1.015e+1	9.839e-1	1.568e+1	1.019e+1	2.327e-1	1.587e+1	2.176e+1
0.07	8.313e+0	2.413e+0	1.211e+1	8.362e+0	1.768e+0	1.230e+1	1.645e+1
0.08	7.094e+0	3.209e+0	9.783e+0	7.154e+0	2.666e+0	9.977e+0	1.299e+1
0.09	6.226e+0	3.637e+0	8.170e+0	6.297e+0	3.200e+0	8.365e+0	1.061e+1
0.10	5.577e+0	3.849e+0	7.001e+0	5.658e+0	3.499e+0	7.197e+0	8.903e+0
0.11	5.068e+0	3.918e+0	6.120e+0	5.159e+0	3.653e+0	6.316e+0	7.629e+0
0.12	4.655e+0	3.899e+0	5.435e+0	4.757e+0	3.708e+0	5.633e+0	6.652e+0
0.13	4.309e+0	3.822e+0	4.889e+0	4.423e+0	3.696e+0	5.087e+0	5.881e+0
0.14	4.014e+0	3.711e+0	4.442e+0	4.136e+0	3.641e+0	4.640e+0	5.263e+0
0.15	3.755e+0	3.577e+0	4.071e+0	3.886e+0	3.556e+0	4.269e+0	4.757e+0
0.16	3.525e+0	3.431e+0	3.755e+0	3.663e+0	3.450e+0	3.952e+0	4.335e+0
0.17	3.318e+0	3.280e+0	3.482e+0	3.462e+0	3.332e+0	3.679e+0	3.979e+0
0.18	3.129e+0	3.128e+0	3.245e+0	3.279e+0	3.207e+0	3.439e+0	3.674e+0
0.19	2.956e+0	2.977e+0	3.035e+0	3.111e+0	3.080e+0	3.227e+0	3.410e+0
0.20	2.796e+0	2.830e+0	2.848e+0	2.954e+0	2.950e+0	3.038e+0	3.178e+0
0.22	2.511e+0	2.554e+0	2.527e+0	2.673e+0	2.698e+0	2.712e+0	2.792e+0
0.24	2.263e+0	2.303e+0	2.262e+0	2.425e+0	2.460e+0	2.439e+0	2.482e+0
0.25	2.151e+0	2.188e+0	2.145e+0	2.313e+0	2.348e+0	2.318e+0	2.348e+0
0.26	2.046e+0	2.080e+0	2.037e+0	2.206e+0	2.241e+0	2.206e+0	2.226e+0
0.28	1.857e+0	1.883e+0	1.844e+0	2.012e+0	2.041e+0	2.004e+0	2.010e+0
0.30	1.690e+0	1.709e+0	1.678e+0	1.838e+0	1.862e+0	1.828e+0	1.826e+0
0.32	1.544e+0	1.557e+0	1.533e+0	1.683e+0	1.702e+0	1.673e+0	1.667e+0
0.34	1.415e+0	1.424e+0	1.406e+0	1.545e+0	1.559e+0	1.536e+0	1.528e+0
0.35	1.357e+0	1.364e+0	1.349e+0	1.482e+0	1.494e+0	1.473e+0	1.465e+0
0.36	1.302e+0	1.307e+0	1.295e+0	1.422e+0	1.432e+0	1.414e+0	1.406e+0
0.38	1.202e+0	1.205e+0	1.196e+0	1.313e+0	1.320e+0	1.306e+0	1.299e+0
0.40	1.113e+0	1.115e+0	1.109e+0	1.215e+0	1.219e+0	1.210e+0	1.203e+0
0.42	1.035e+0	1.035e+0	1.032e+0	1.127e+0	1.130e+0	1.124e+0	1.118e+0
0.44	9.651e-1	9.649e-1	9.636e-1	1.049e+0	1.051e+0	1.046e+0	1.042e+0
0.45	9.332e-1	9.327e-1	9.320e-1	1.013e+0	1.014e+0	1.011e+0	1.007e+0
0.46	9.030e-1	9.023e-1	9.021e-1	9.793e-1	9.799e-1	9.773e-1	9.740e-1
0.48	8.474e-1	8.465e-1	8.471e-1	9.164e-1	9.164e-1	9.152e-1	9.127e-1
0.50	7.975e-1	7.965e-1	7.975e-1	8.599e-1	8.595e-1	8.592e-1	8.574e-1
0.55	6.928e-1	6.919e-1	6.934e-1	7.415e-1	7.407e-1	7.415e-1	7.411e-1
0.60	6.101e-1	6.094e-1	6.108e-1	6.484e-1	6.476e-1	6.488e-1	6.490e-1
0.65	5.432e-1	5.427e-1	5.438e-1	5.738e-1	5.732e-1	5.743e-1	5.748e-1
0.70	4.878e-1	4.874e-1	4.883e-1	5.129e-1	5.124e-1	5.134e-1	5.140e-1
0.80	4.009e-1	4.007e-1	4.012e-1	4.194e-1	4.192e-1	4.198e-1	4.203e-1
0.90	3.355e-1	3.354e-1	3.357e-1	3.506e-1	3.505e-1	3.508e-1	3.512e-1
1.00	2.843e-1	2.843e-1	2.845e-1	2.976e-1	2.975e-1	2.977e-1	2.980e-1
1.10	2.434e-1	2.433e-1	2.435e-1	2.554e-1	2.553e-1	2.555e-1	2.556e-1
1.20	2.100e-1	2.100e-1	2.101e-1	2.211e-1	2.211e-1	2.212e-1	2.213e-1

1.30	1.827e-1	1.826e-1	1.827e-1	1.929e-1	1.928e-1	1.929e-1	1.930e-1
1.40	1.599e-1	1.599e-1	1.600e-1	1.694e-1	1.693e-1	1.694e-1	1.694e-1
1.50	1.410e-1	1.410e-1	1.410e-1	1.496e-1	1.496e-1	1.497e-1	1.497e-1
1.60	1.250e-1	1.250e-1	1.251e-1	1.330e-1	1.330e-1	1.330e-1	1.330e-1
1.70	1.116e-1	1.115e-1	1.116e-1	1.188e-1	1.188e-1	1.188e-1	1.188e-1
1.80	1.001e-1	1.001e-1	1.001e-1	1.067e-1	1.067e-1	1.067e-1	1.067e-1
1.90	9.024e-2	9.024e-2	9.025e-2	9.629e-2	9.628e-2	9.629e-2	9.630e-2
2.00	8.177e-2	8.177e-2	8.177e-2	8.730e-2	8.729e-2	8.730e-2	8.730e-2
2.50	5.314e-2	5.314e-2	5.314e-2	5.674e-2	5.674e-2	5.674e-2	5.674e-2
3.00	3.738e-2	3.738e-2	3.738e-2	3.986e-2	3.987e-2	3.986e-2	3.986e-2
3.50	2.779e-2	2.779e-2	2.779e-2	2.960e-2	2.960e-2	2.960e-2	2.960e-2
4.00	2.149e-2	2.149e-2	2.149e-2	2.288e-2	2.288e-2	2.288e-2	2.288e-2
5.00	1.397e-2	1.397e-2	1.397e-2	1.486e-2	1.486e-2	1.486e-2	1.486e-2
6.00	9.794e-3	9.795e-3	9.794e-3	1.043e-2	1.043e-2	1.043e-2	1.043e-2

Table 2.

Parameterization of X-ray scattering factors of ions for the 4 Gaussians + a constant model

ion	a_1	a_2	a_3	a_4	c	R_{scat} *
	b_1	b_2	b_3	b_4		(%)
C^+	6.727e+0	1.439e+0	2.191e+0	9.913e-1	-6.349e+0	0.0154
	4.479e-3	6.231e-1	1.313e+1	2.971e+1		
C^-	1.598e+0	2.161e+0	2.360e+0	6.549e-1	2.238e-1	0.0606
	5.843e-1	1.346e+1	3.831e+1	1.405e+2		
N^+	1.570e+0	2.068e+0	1.847e+0	2.870e-1	2.271e-1	0.0025
	4.122e-1	8.064e+0	1.665e+1	3.340e+1		
N^-	1.569e+0	2.640e+0	2.795e+0	7.386e-1	2.551e-1	0.0394
	4.393e-1	9.202e+0	2.624e+1	9.062e+1		
O^+	1.553e+0	2.237e+0	2.466e+0	5.079e-1	2.367e-1	0.0015
	3.148e-1	5.618e+0	1.191e+1	2.504e+1		
O^-	1.535e+0	3.038e+0	3.280e+0	8.555e-1	2.886e-1	0.0346
	3.459e-1	6.556e+0	1.876e+1	6.328e+1		
P^+	1.732e+0	6.472e+0	3.698e+0	9.940e-1	1.105e+0	0.0065
	5.089e-1	1.893e+0	2.424e+1	5.215e+1		
P^-	1.990e+0	6.243e+0	4.686e+0	1.928e+0	1.146e+0	0.0423
	5.903e-1	1.967e+0	3.046e+1	1.031e+2		
P^{2+}	1.825e+0	6.368e+0	3.076e+0	6.076e-1	1.124e+0	0.0046
	5.431e-1	1.924e+0	2.114e+1	4.185e+1		
S^+	1.419e+0	6.923e+0	4.736e+0	1.076e+0	8.458e-1	0.0115
	2.405e-1	1.459e+0	2.028e+1	4.519e+1		
S^-	1.505e+0	6.721e+0	5.614e+0	2.130e+0	1.024e+0	0.0372
	3.506e-1	1.507e+0	2.411e+1	7.728e+1		
S^{2+}	1.442e+0	6.787e+0	4.206e+0	5.939e-1	9.715e-1	0.0092
	3.088e-1	1.488e+0	1.846e+1	3.950e+1		
S^{3+}	1.611e+0	6.550e+0	3.434e+0	3.284e-1	1.078e+0	0.0051
	4.062e-1	1.543e+0	1.637e+1	3.422e+1		

* Defined in equation (6). Calculated between the scattering factors and the fitted curves. Summation over data from $\sin\theta/\lambda = 0$ to 2 $Å^{-1}$.

Table 3.

Parameterization of X-ray scattering factors of ions for the 5 Gaussians + a constant model

ion	a_1 / b_1	a_2 / b_2	a_3 / b_3	a_4 / b_4	a_5 / b_5	c	R_{scat} * (%)
C^+	8.431e-2	5.957e-1	1.145e+0	2.224e+0	9.528e-1	-2.457e-3	0.0194
	4.787e-2	2.273e-1	7.042e-1	1.328e+1	3.012e+1		
C^-	5.946e-1	1.229e+0	2.295e+0	2.257e+0	6.050e-1	1.642e-2	0.0516
	1.840e-1	7.149e-1	1.408e+1	4.041e+1	1.463e+2		
N^+	1.670e-1	6.515e-1	9.987e-1	2.749e+0	1.427e+0	5.943e-3	0.0228
	6.426e-2	2.094e-1	5.482e-1	9.105e+0	2.215e+1		
N^-	6.423e-1	1.169e+0	2.685e+0	2.758e+0	7.215e-1	2.224e-2	0.0403
	1.451e-1	5.304e-1	9.338e+0	2.664e+1	9.161e+1		
O^+	6.042e-1	1.171e+0	2.398e+0	2.376e+0	4.253e-1	2.527e-2	0.0047
	1.078e-1	3.764e-1	5.797e+0	1.247e+1	2.613e+1		
O^-	6.950e-1	1.102e+0	3.023e+0	3.287e+0	8.611e-1	2.919e-2	0.0367
	1.195e-1	4.139e-1	6.546e+0	1.869e+1	6.309e+1		
P^+	1.496e+0	1.992e+0	5.676e+0	3.739e+0	9.422e-1	1.546e-1	0.0107
	7.120e-2	9.188e-1	1.987e+0	2.446e+1	5.304e+1		
P^-	1.505e+0	2.539e+0	5.185e+0	4.696e+0	1.909e+0	1.595e-1	0.0431
	7.246e-2	1.008e+0	2.097e+0	3.061e+1	1.036e+2		
P^{2+}	1.501e+0	2.253e+0	5.421e+0	3.145e+0	5.236e-1	1.563e-1	0.0084
	7.155e-2	9.661e-1	2.041e+0	2.148e+1	4.363e+1		
S^+	1.477e+0	1.268e+0	6.299e+0	4.788e+0	1.011e+0	1.569e-1	0.0155
	6.175e-2	6.681e-1	1.521e+0	2.047e+1	4.613e+1		
S^-	1.483e+0	1.597e+0	6.016e+0	5.623e+0	2.114e+0	1.610e-1	0.0397
	6.259e-2	7.320e-1	1.570e+0	2.419e+1	7.758e+1		
S^{2+}	1.483e+0	1.520e+0	6.052e+0	4.257e+0	5.300e-1	1.594e-1	0.0131
	6.220e-2	7.229e-1	1.558e+0	1.865e+1	4.092e+1		
S^{3+}	1.489e+0	1.983e+0	5.618e+0	3.476e+0	2.716e-1	1.626e-1	0.0084
	6.277e-2	8.002e-1	1.631e+0	1.656e+1	3.620e+1		

* Defined in equation (6). Calculated between the scattering factors and the fitted curves. Summation over data from sin θ / λ = 0 to 6 $Å^{-1}$.

Table 4.

Parameterization of electron scattering factors of ions for the 5 Gaussians + a charge term model

ion	a_1<br>b_1	a_2<br>b_2	a_3<br>b_3	a_4<br>b_4	a_5<br>b_5	R_{scat} *<br>(%)
C^+	2.079e-2	9.266e-2	2.949e-1	6.812e-1	3.304e-1	0.0156
	5.950e-2	5.359e-1	2.760e+0	9.283e+0	2.442e+1	
C^-	2.248e-1	8.254e-1	1.769e+0	1.690e+0	6.994e-1	0.0810
	5.518e-1	4.308e+0	1.600e+1	5.196e+1	1.708e+2	
N^+	2.296e-2	1.004e-1	3.289e-1	6.546e-1	2.733e-1	0.0132
	5.522e-2	4.910e-1	2.402e+0	7.751e+0	2.051e+1	
N^-	2.192e-1	7.256e-1	1.398e+0	1.245e+0	4.381e-1	0.0430
	4.784e-1	3.389e+0	1.171e+1	3.604e+1	1.125e+2	
O^+	2.439e-2	1.036e-1	3.360e-1	6.112e-1	2.447e-1	0.0120
	5.082e-2	4.390e-1	2.036e+0	6.407e+0	1.710e+1	
O^-	2.236e-1	6.923e-1	1.176e+0	9.354e-1	2.821e-1	0.0218
	4.372e-1	2.918e+0	9.670e+0	2.868e+1	8.489e+1	
P^+	6.191e-2	3.154e-1	6.324e-1	1.661e+0	1.027e+0	0.0447
	6.525e-2	6.224e-1	3.009e+0	1.258e+1	3.411e+1	
P^-	4.590e-1	1.002e+0	2.724e+0	3.228e+0	1.336e+0	0.0728
	5.019e-1	3.545e+0	1.445e+1	4.313e+1	1.303e+2	
P^{2+}	4.997e-2	2.497e-1	4.690e-1	1.099e+0	6.144e-1	0.0233
	5.712e-2	5.372e-1	2.398e+0	1.051e+1	2.704e+1	
S^+	6.232e-2	3.129e-1	6.541e-1	1.742e+0	9.377e-1	0.0391
	6.149e-2	5.785e-1	2.848e+0	1.107e+1	2.978e+1	
S^-	4.496e-1	9.810e-1	2.598e+0	2.717e+0	8.614e-1	0.0543
	4.656e-1	3.259e+0	1.233e+1	3.583e+1	1.055e+2	
S^{2+}	5.029e-2	2.440e-1	4.671e-1	1.196e+0	7.100e-1	0.0230
	5.364e-2	4.908e-1	2.186e+0	8.911e+0	2.266e+1	
S^{3+}	3.991e-2	1.787e-1	3.465e-1	7.425e-1	5.600e-1	0.0124
	4.652e-2	4.001e-1	1.626e+0	6.936e+0	1.724e+1	

* Defined in equation (6). Calculated between the scattering factors and the fitted curves. Summation over data from sin θ/λ = 0.04 to 6 $Å^{-1}$ for the positive ions and from 0.04 to 1.5 $Å^{-1}$ for the negative ions.
